# Socioeconomic Inequalities in COVID-19 Vaccination and Infection in Adults, Catalonia, Spain

**DOI:** 10.3201/eid2811.220614

**Published:** 2022-11

**Authors:** Elena Roel, Berta Raventós, Edward Burn, Andrea Pistillo, Daniel Prieto-Alhambra, Talita Duarte-Salles

**Affiliations:** Universitat Autònoma de Barcelona, Barcelona, Spain (E. Roel, B. Raventós);; Fundació Institut Universitari per a la recerca a l’Atenció Primària de Salut Jordi Gol i Gurina, Barcelona, Spain (E. Roel, B. Raventós, E. Burn, A. Pistillo, T. Duarte-Salles);; Centre for Statistics in Medicine, Nuffield Department of Orthopaedics, Rheumatology, and Musculoskeletal Sciences, University of Oxford, Oxfordshire, United Kingdom (E. Burn, D. Prieto-Alhambra);; Erasmus University Medical Center Department of Medical Informatics, Rotterdam, Netherlands (D. Prieto-Alhambra)

**Keywords:** COVID-19, SARS-CoV-2, COVID-19 vaccines, severe acute respiratory syndrome coronavirus 2, viruses, respiratory infections, zoonoses, social class, socioeconomic factors, health inequities, Spain, electronic health records, coronavirus disease

## Abstract

Evidence on the impact of the COVID-19 vaccine rollout on socioeconomic COVID-19–related inequalities is scarce. We analyzed associations between socioeconomic deprivation index (SDI) and COVID-19 vaccination, infection, and hospitalization before and after vaccine rollout in Catalonia, Spain. We conducted a population-based cohort study during September 2020–June 2021 that comprised 2,297,146 adults >40 years of age. We estimated odds ratio of nonvaccination and hazard ratios (HRs) of infection and hospitalization by SDI quintile relative to the least deprived quintile, Q1. Six months after rollout, vaccination coverage differed by SDI quintile in working-age (40–64 years) persons: 81% for Q1, 71% for Q5. Before rollout, we found a pattern of increased HR of infection and hospitalization with deprivation among working-age and retirement-age (>65 years) persons. After rollout, infection inequalities decreased in both age groups, whereas hospitalization inequalities decreased among retirement-age persons. Our findings suggest that mass vaccination reduced socioeconomic COVID-19–related inequalities.

The COVID-19 pandemic has caused an unprecedented global health crisis, resulting in >540 million cases worldwide as of July 2022 (*1*). However, the impact of the pandemic has not been uniform across or within countries (*2*). Disadvantaged populations, such as individuals with low socioeconomic status, display higher incidence rates of COVID-19 infection and hospitalization (*3*,*4*). To date, vaccines against SARS‑CoV‑2, the virus that causes COVID-19, are the cornerstone of the COVID-19 response. Yet, emerging evidence shows socioeconomic inequalities in COVID-19 vaccination coverage within countries with high access to vaccines, such as the United Kingdom or the United States (*5*–*8*). For instance, a report from May 2021 from the United Kingdom showed that vaccination coverage was 94% in the least areas and 84% in the most deprived areas (deprivation was measured using an index based on income, employment, education, health, crime, barriers to housing and services, and living environment) (*8*,*9*). Similarly, in the United States, vaccination coverage was lower (49%) among adults living in counties with the highest overall social vulnerability index (SVI) scores (based on socioeconomic status, household composition and disability, racial/ethnic minority status and language, and housing type and transportation) when compared to the coverage (59%) among adults living in counties with the lowest overall SVI scores in May 2021 (*10*). However, evidence is scarce regarding socioeconomic inequalities in COVID-19 vaccine uptake from other countries and the effect of the COVID-19 vaccine rollout on socioeconomic COVID-19–related outcomes inequalities.

In Spain, the COVID-19 vaccine rollout started on December 27, 2020. The first population groups eligible for vaccination were persons living in nursing homes and healthcare workers (*11*). Subsequently, other groups became eligible, taking into account age, starting with the eldest; underlying conditions, prioritizing persons with risk factors for COVID-19; and occupation, prioritizing essential workers. In Catalonia, a region located in northeast Spain, 52% of the population had received >1 dose of a COVID-19 vaccine as of June 30, 2021 (*12*). Determining patterns of socioeconomic inequalities in relation to COVID-19 vaccination and COVID-19 outcomes in Catalonia could provide valuable information to public health authorities to guide immunization efforts among vulnerable populations in Spain and in other countries with widespread access to vaccines.

We analyzed the association between a socioeconomic deprivation index (SDI) score based on place of residence (a proxy measure of socioeconomic status) and COVID-19 vaccination coverage 6 months after the start of vaccine rollout among adults >40 years of age living in urban areas of Catalonia. Subsequently, we analyzed the associations between SDI score and COVID-19 infection, hospitalization, and death, before and after the start of vaccine rollout. The Clinical Research Ethics committee of Fundació Institut Universitari per a la recerca a l’Atenció Primària de Salut Jordi Gol i Gurina (IDIAPJGol) approved this study (project code 21/052-PCV), with no required written consent from participants.

## Methods

### Study Design and Data Source

We conducted a population-based cohort study during September 1, 2020–June 30, 2021, using primary care data from the Information System for Research in Primary Care (SIDIAP; https://www.sidiap.org) database, standardized to the Observational Medical Outcomes Partnership Common Data Model (*13*,*14*). SIDIAP contains pseudoanonymized electronic health records from ≈75% of the population in Catalonia, which has ≈7.5 million inhabitants, and is representative in terms of age, sex, and geographic distribution (*15*). SIDIAP includes data on sociodemographics, diagnoses, laboratory tests, medication use, and deaths. In addition, SIDIAP has been linked to the Catalan public health vaccine registry and to a population-based register of hospital discharge records from public and private hospitals of Catalonia (Conjunt Mínim Bàsic de Dades d’Alta Hospitalària, CMBD-AH) (E. Burn, et al., unpub. data, https://doi.org/10.1101/2021.11.23.21266734).

### Study Participants

**Figure 1:**
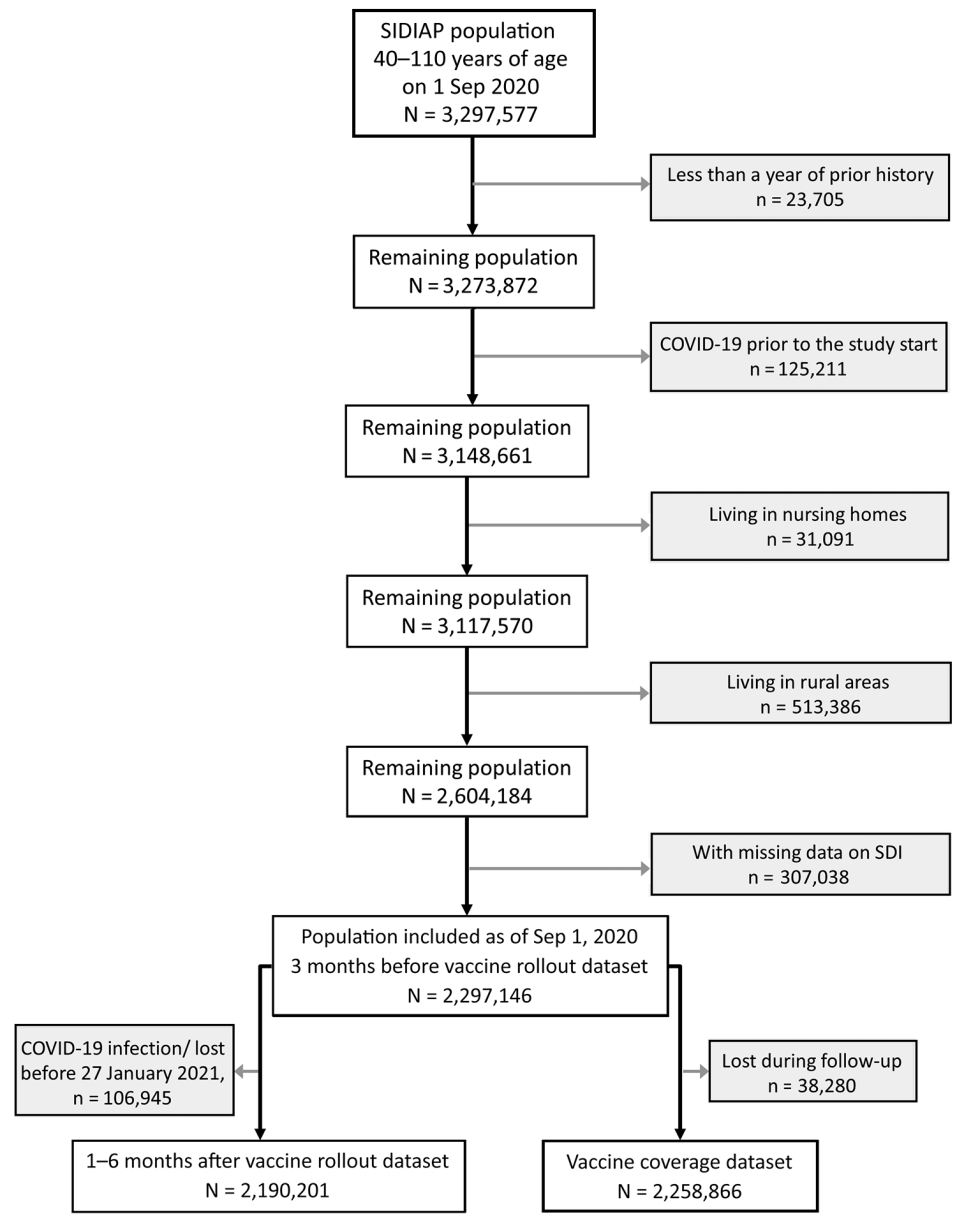
Flowchart showing the inclusion and exclusion criteria for study population in analysis of socioeconomic inequalities in COVID-19 vaccination and infection in adults, Catalonia, Spain. SDI, Socioeconomic Deprivation Index; SIDIAP, Information System for Research in Primary Care.

We included 2,297,146 adults 40–110 years of age registered in SIDIAP as of September 1, 2020, after excluding those with <1 year of medical history available (n = 23,705), those with a previous COVID-19 infection (n = 125,111), those living in nursing homes (n = 31,091) and in rural areas (n = 513,386), and those with missing data on SDI (n = 307,038) (Figure 1). We included adults >40 years of age because those younger were not generally eligible for vaccination before mid-June 2021. We excluded persons living in rural areas, which included municipalities with <10,000 inhabitants and a population density <150 habitants/km^2^ (*16*), because information on SDI was unavailable for these areas. We identified persons with a previous COVID-19 infection using SARS-CoV-2 positive tests or clinical COVID-19 diagnoses because SARS-CoV-2 tests were restricted to severe cases during the first months of the pandemic in Spain (*17*). We used Systematized Nomenclature of Medicine codes to identify COVID-19 diagnoses (Appendix Table 1).

To assess inequalities in COVID-19 vaccination coverage 6 months after the start of vaccine rollout (i.e., June 30, 2021), we restricted our analyses to persons with complete follow-up (vaccine coverage dataset, n = 2,258,866). We analyzed inequalities on COVID-19 outcomes for 2 time periods: 3 months before and 1–6 months after the start of vaccine rollout. For each period, we followed participants until the occurrence of the outcome of interest, end of study period, exit from database, or death, whichever occurred first. The period 3 months before vaccine rollout was September 1–December 26, 2020. The period 1–6 months after vaccine rollout was January 27–June 30, 2021; we excluded patients with a COVID-19 infection or lost before January 27, 2021 (n = 106,945), from analysis.

### Outcomes

We identified persons vaccinated against COVID-19 as those who had received a dose of any COVID-19 vaccine: BNT162b2 mRNA (Pfizer-BioNTech, https://www.pfizer.com), mRNA-1273 (Moderna, https://www.modernatx.com), ChAdOx1 nCoV-19 (Oxford-AstraZeneca, https://www.astrazeneca.com), or Ad.26.COV2.S (Janssen/J&J, https://www.janssen.com). The date of vaccination was the date of the first dose administration. We identified COVID-19 infections based on a positive SARS-CoV-2 antigen or reverse transcription PCR test, using the test date as the date of infection; we considered the first infection per person. We defined COVID-19 hospitalizations as hospitalizations with a positive SARS-CoV-2 test result between 21 days before and 3 days after the date of admission. We defined COVID-19–related deaths as deaths occurring <28 days after the date of infection.

### Variables

We measured SDI score using the Mortalidad en áreas pequeñas españolas y desigualdades socioeconómicas y ambientales (MEDEA) deprivation index (*16*). The MEDEA index was calculated for census tract urban areas using information related to 5 indicators (related to work and education) from the 2001 national census in Spain. We linked the MEDEA deprivation index to each participant’s most recent site of residence and categorized it into quintiles of socioeconomic deprivation, with the first quintile (Q1) representing the least deprived and the fifth (Q5) the most deprived area. We extracted age in years, sex, nationality by the country’s geographic region, and comorbidities recorded before study start that were identified using Systematized Nomenclature of Medicine codes (Appendix Table 1). We categorized age into 2 groups: >65 (retirement age) and 40–64 years (working age).

### Statistical Analysis

We described participants’ characteristics at baseline and by vaccination status, COVID-19 infection, hospitalization, and death over study follow-up period; we used counts and percentages for categorical variables and median and interquartile ranges (IQRs) for continuous variables. In accordance with information-governance requirements intended to protect confidentiality, we reported results with <5 persons as <5 rather than specific numbers. We also compared baseline characteristics of persons with and without missing data on SDI, and those with and without complete follow-up, using standardized mean differences (SMD). We considered an absolute SMD >0.1 to be a meaningful difference in the distribution of a given characteristic between the groups compared (*18*). We generated charts of weekly cumulative vaccination coverages and incidence rates (IRs; cases/100,000 person-years) of COVID-19 infection, hospitalization, and death during September 1, 2020–June 30, 2021, by SDI quintile and age group. We used R version 4.1 (The R Project for Statistical Computing, https://www.r-project.org) for data curation, analysis, and visualization.

To assess the association between SDI quintile and nonvaccination, we performed crude and adjusted logistic regression models and calculated odds ratios (ORs) with 95% CIs by age group. We included persons with complete follow-up for these analyses (vaccine coverage dataset). To assess the association between SDI quintile and COVID-19 infection, hospitalization and death, we performed crude and adjusted Cox proportional-hazards models and calculated hazard ratios (HRs) with 95% CIs by age group and period using the 3 months before and 1–6 months after vaccine rollout datasets. We visually inspected log-log survival curves to check the proportional hazard assumptions for the variables included in the models. We did not estimate models in which the number of events per SDI quintile was <5. Models were relative to the least deprived quintile (Q1) and adjusted by age, sex, and nationality; we developed a directed acyclic graph to guide our modeling strategy (Appendix Figure 1) (*19*). Of note, rates of hospitalization and death were estimated among the total population rather than among those infected with COVID-19 to prevent collider bias (*20*).

In addition, we performed 3 sensitivity analyses. First, we reestimated our models for vaccination coverage after excluding persons with a COVID-19 infection during follow-up, because they were not eligible for vaccination until 6 months after the infection. Second, we reestimated our models for COVID-19 outcomes restricting our analyses to citizens of Spain because the proportionality assumption was violated for nationality and all the COVID-19 outcomes. Third, we estimated socioeconomic inequalities on COVID-19 outcomes for the time period 3–6 months after the start of vaccine rollout, March 27– June 30, 2021, after excluding those with a COVID-19 infection, deceased, or lost before March 27, 2021 (n = 137,663).

## Results

Among the 2,297,146 participants included, most (n = 1,518,851; 66.1%) were 40–64 years of age (median 57 years of age), were citizens of Spain (88.8%), and had few comorbidities (Table). Persons living in more deprived areas were younger, less frequently citizens of Spain, and had more comorbidities than those living in the least deprived ones (Appendix Table 2). Persons excluded because of missing data on SDI were slightly younger (median age 55 years), more frequently from Europe and North America, and less frequently from Asia and Oceania than those without missing data on SDI (Appendix Table 3). Compared with those in the vaccine coverage dataset (i.e., with complete follow-up), persons with incomplete follow-up (lost to follow-up) (n = 38,280; 1.7%) were older (median age 69 years), were less frequently citizens of Spain (80.3%), and had more comorbidities (Appendix Table 4). For 51.5% of that population, death was the reason patients were lost to follow-up.

**Table:** Population characteristics in study of socioeconomic inequalities in COVID-19 vaccination and infection, Catalonia, Spain, 2020–2021*

Characteristic	Population	Vaccinated†	Infected with COVID-19	Hospitalized with COVID-19	COVID-19–related death
Total	2,297,146 (100.0)	1,852,361 (82.0)	134,966 (5.9)	16,921 (0.7)	1,881 (0.1)
Loss to follow-up	38,280 (1.7)	0	3,580 (9.4)	1,779 (4.6)	1,881 (4.9)
Median age, y (IQR)	57 (48–69)	59 (49–71)	54 (47–66)	66 (55–77)	84 (76–89)
Age category, y					
40–49	694,924 (30.3)	481,716 (70.2)	47,121 (6.8)	2,330 (0.3)	10 (0.0)
50–59	582,558 (25.4)	473,371 (82.1)	38,092 (6.5)	3,530 (0.6)	64 (0.0)
60–69	450,173 (19.6)	385,155 (86.6)	23,525 (5.2)	3,815 (0.8)	162 (0.0)
70–79	345,152 (15.0)	316,244 (93.2)	15,221 (4.4)	3,793 (1.1)	402 (0.1)
>80	224,339 (9.8)	195,875 (92.6)	11,007 (4.9)	3,453 (1.5)	1,243 (0.6)
Sex					
F	1,200,296 (52.3)	987,415 (83.5)	71,185 (5.9)	7,262 (0.6)	802 (0.1)
M	1,096,850 (47.7)	864,946 (80.4)	63,781 (5.8)	9,659 (0.9)	1,079 (0.1)
Nationality					
Spain	2,040,130 (88.8)	1,726,192 (85.9)	117,423 (5.8)	14,864 (0.7)	1,833 (0.1)
Africa	69,086 (3.0)	30,053 (44.8)	5,161 (7.5)	580 (0.8)	13 (0.0)
Central & South America	70,312 (3.1)	40,287 (59.2)	6,368 (9.1)	740 (1.1)	10 (0.0)
Asia & Oceania	47,906 (2.1)	21,888 (46.9)	3,063 (6.4)	443 (0.9)	10 (0.0)
Eastern Europe	34,803 (1.5)	13,015 (38.4)	1,674 (4.8)	177 (0.5)	<5
Western Europe & North America	34,909 (1.5)	20,926 (61.9)	1,277 (3.7)	117 (0.3)	12 (0.0)
SDI quintile					
Q1	478,380 (20.8)	397,672 (84.6)	25,441 (5.3)	2,748 (0.6)	345 (0.1)
Q2	469,833 (20.5)	387,994 (83.8)	26,302 (5.6)	3,123 (0.7)	370 (0.1)
Q3	465,245 (20.3)	378,990 (82.7)	26,955 (5.8)	3,393 (0.7)	389 (0.1)
Q4	453,924 (19.8)	364,672 (81.7)	27,154 (6.0)	3,604 (0.8)	382 (0.1)
Q5	429,764 (18.7)	323,033 (76.7)	29,114 (6.8)	4,053 (0.9)	395 (0.1)
Comorbidities					
Asthma	141,725 (6.2)	118,256 (84.7)	9,344 (6.6)	1,373 (1.0)	134 (0.1)
Autoimmune disease	58,146 (2.5)	50,527 (88.6)	3,470 (6.0)	607 (1.0)	101 (0.2)
COPD	119,845 (5.2)	105,236 (91.4)	6,497 (5.4)	1,999 (1.7)	403 (0.3)
Dementia	30,223 (1.3)	24,747 (92.7)	2,082 (6.9)	597 (2.0)	314 (1.0)
Heart disease	402,389 (17.5)	353,597 (90.8)	22,829 (5.7)	5,696 (1.4)	1,172 (0.3)
Hypertension	775,420 (33.8)	681,602 (90.0)	43,425 (5.6)	9,319 (1.2)	1,498 (0.2)
Obesity	515,509 (22.4)	435,893 (85.9)	34,914 (6.8)	6,619 (1.3)	626 (0.1)
Malignant neoplastic disease	264,658 (11.5)	233,327 (91.4)	14,285 (5.4)	3,165 (1.2)	677 (0.3)
Renal impairment	169,947 (7.4)	149,516 (92.5)	9,690 (5.7)	3,131 (1.8)	840 (0.5)
Type 2 diabetes	288,188 (12.5)	251,117 (89.7)	17,689 (6.1)	4,637 (1.6)	758 (0.3)

*Values are no. (%) except as indicated. Study population as of September 1, 2020, We noted characteristics overall and by vaccination, COVID-19 infection, hospitalization, and death status over follow-up period. Quintiles listed from least deprived (Q1) to most deprived (Q5). COPD, chronic obstructive pulmonary disease; IQR, interquartile range; SDI, Socioeconomic Deprivation Index. †Among those with complete follow-up, n = 2,258,866.

### Vaccination Coverage and COVID-19 Infections, Hospitalizations, and Deaths at Study End

Six months after vaccine rollout, among those with complete follow-up (n = 2,258,866), 82.0% had been vaccinated. Vaccination coverage was highest among older persons (>80 years; 92.6%), women (83.5%), those living in the least deprived areas (84.6% for Q1 vs. 76.7% for Q5), and those with comorbidities (e.g., 92.7% among persons with dementia) (Table). Vaccination coverage was particularly low among persons of other nationality: ≈60% for those from western Europe and America and <50% for those from Africa, Asia, and Oceania and from eastern Europe.

During September 1, 2020–June 30, 2021, a total of 134,966 (5.9%) persons were infected with COVID-19; of those, 16,921 (0.7%) were hospitalized for COVID-19, and 1,881 (0.1%) died (Table). Cases of COVID-19 were highest among younger persons, 40–49 years of age (6.8%), followed by those >80 years of age (4.9%); COVID-19 was also more common among migrants from Central and South America (9.1%) and Africa (7.5%) than for citizens of Spain (5.8%) and in the most deprived areas (6.8% for Q5) than the least deprived (5.3% for Q1). Conversely, hospitalizations were highest among the eldest (>80 years; 1.5%), men (0.9%), those from Central and South America (1.1%), those with comorbidities (e.g., 1.8% among those with renal impairment), and those from the most deprived areas (0.9% for Q5 vs. 0.6% for Q1). Death rates were overall similar by sex, nationality, and SDI quintile but were higher among the eldest (0.6%) and those with comorbidities.

### Trends in Vaccination Coverage and COVID-19 Infection, Hospitalization, and Death over Time

**Figure 2:**
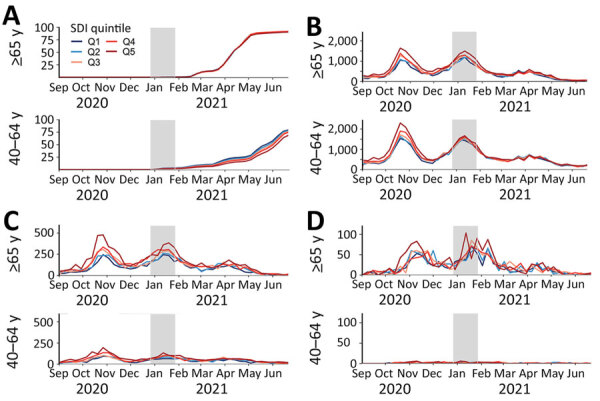
Vaccination coverage and incidence rates of COVID-19 infection, hospitalization, and death over time by SDI quintile and age group in study of socioeconomic inequalities in COVID-19 vaccination and infection, Catalonia, Spain, 2020–2021. Only persons with complete follow-up were included to estimate vaccination coverages. Gray area shows the first month after the start of vaccine rollout (December 27, 2020). Q1 represents the least deprived quintile, Q5 the most deprived. A) Vaccination coverage by age group, shown as percentage of population. B) COVID-19 infections by age group, shown as incidence rate per 100,000 person-years. C) COVID-19 hospitalizations, shown as incidence rate per 100,000 person-years. COVID-19–related deaths, shown as incidence rate per 100,000 person-years. Q, quintile; SDI, Socioeconomic Deprivation Index.

Among participants >65 years of age, vaccination coverage over time was similar across all SDI quintiles, whereas in those 40–64 years of age we observed a pattern of lower vaccination coverage in areas with increased socioeconomic deprivation (Figure 2). Regarding COVID-19 outcomes, IR of infection peaked in mid-October 2020 and mid-January 2021 and plateaued after March 2021. We observed a similar pattern for COVID-19 hospitalizations and deaths. Infection rates were higher among those 40–64 years of age, whereas hospitalization and death rates were higher among those ≥65 years of age. Overall, we observed a pattern of higher IR of infection and hospitalization in areas with increased socioeconomic deprivation among both age groups for the IR peaks. As for COVID-19 deaths, we found those living in the most deprived areas had the the higher IR for those peaks, without a clear pattern of increased IR with increased socioeconomic deprivation. After March 2021, differences by SDI quintile for all COVID-19 outcomes were less obvious, because IR of infection, hospitalization, and death were much lower.

### Associations between SDI Quintile and Nonvaccination

**Figure 3:**
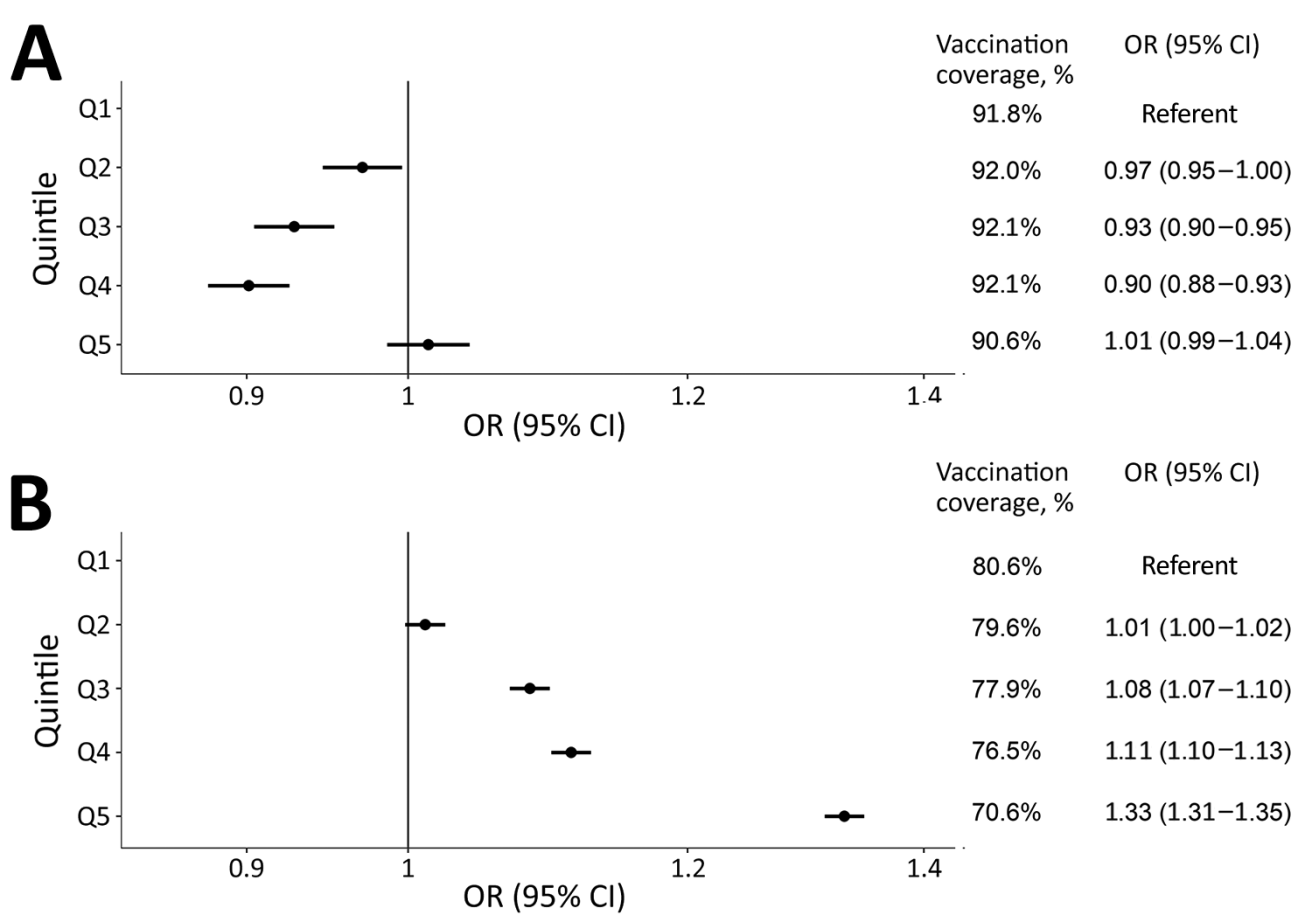
Odds ratios of nonvaccination 6 months after the start of COVID-19 vaccine rollout by Socioeconomic Deprivation Index quintile, stratified by age group, in study of socioeconomic inequalities in COVID-19 vaccination and infection, Catalonia, Spain, 2020–2021. A) OR for retirement-age persons >65 years of age. B) OR for working-age persons 40–64 years of age. Q1, the referent quintile, represents the least deprived areas; Q5, the most deprived. Persons with complete follow-up (n = 2,258,866) after vaccination were included. Models are adjusted for age, sex, and nationality. Dots indicate OR; bars, 95% CI. OR, odds ratio; Q, quintile.

Compared with persons >65 years of age living in the least deprived areas (Q1), those living in Q2, Q3, and Q4 areas had a lower probability of nonvaccination. In Q2 areas, OR was 0.97 (95% CI 0.95–1.00); in Q3 areas, 0.93 (95% CI 0.90–0.95); in Q4 areas, 0.90 (95% CI 0.88–0.93); and in Q5 areas, 1.01 (95% CI 0.99–1.04) (Figure 3; Appendix Figure 2). Conversely, among those 40–64 years of age, we found increased odds of nonvaccination for persons living in more deprived areas. For instance, when compared with those living in Q1 areas, OR of nonvaccination was 1.01 (95% CI 1.00–1.02) in Q2 areas, 1.08 (95% CI 1.07–1.10) in Q3 areas, 1.11 (95% CI 1.10–1.13) in Q4 areas, and 1.33 (95% CI 1.31–1.35) in Q5 areas. Sensitivity analyses excluding persons with a COVID-19 infection before vaccination (n = 124,522) were consistent with our main analyses (Appendix Figure 3).

### Association between SDI Quintile and COVID-19 Outcomes

**Figure 4:**
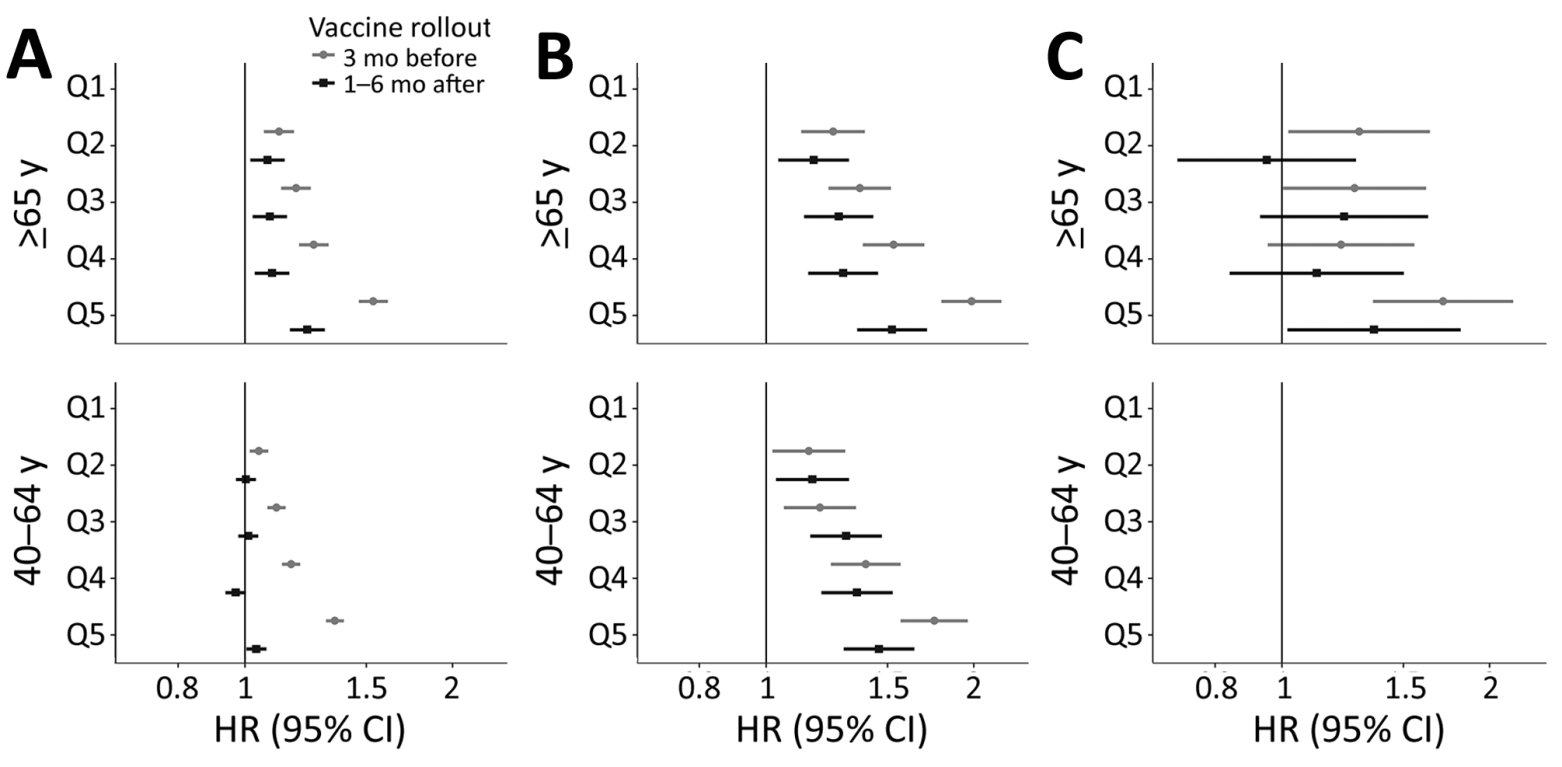
Fully adjusted hazard ratios of COVID-19 infection (A), hospitalization (B), and death (C) before and after vaccine rollout, by Socioeconomic Deprivation Index quintile and stratified by age group, in study of socioeconomic inequalities in COVID-19 vaccination and infection, Catalonia, Spain, 2020–2021. Q1, the referent quintile, represents the least deprived areas; Q5, the most deprived. Vaccine rollout started on December 27, 2020. Models before vaccine rollout are from September 1–December 26, 2020. Models after vaccine rollout are from January 27–June 30, 2021. All models are adjusted for age, sex, and nationality. Models in which the number of events for >1 deprivation area was <5 were not estimated. Dots indicate OR; bars, 95% CI. Q, quintile. HR, hazard ratio.

Three months before vaccine rollout, we observed a pattern of increased HR of COVID-19 infection in more deprived areas in both age groups (Figure 4; Appendix Table 5). For example, among those >65 years of age, HR was 1.12 (95% CI 1.07–1.18) for those living in Q2 areas, 1.19 (95% CI 1.13–1.25) in Q3 areas, 1.26 (95% CI 1.20–1.32) in Q4 areas, and 1.54 (95% CI 1.46–1.61) in Q5 areas. A similar pattern was seen for COVID-19 hospitalizations among both age groups, with larger inequalities. Among persons >65 years of age, HR was 1.25 (95% CI 1.12–1.39) for those living in Q2 areas, 1.37 (95% CI 1.23–1.52) in Q3 areas, 1.53 (95% CI 1.38–1.70) in Q4 areas, and 1.99 (95% CI 1.80–2.19) in Q5 areas. Conversely, this pattern was not apparent for COVID-19–related deaths among persons >65 years of age; rates were only higher for those living in Q5 areas (HR 1.71 [95% CI 1.36–2.17]). We did not estimate models for death among persons 40–64 years of age because we observed <5 events in some SDI quintiles.

In the period 1–6 months after vaccine rollout, inequalities decreased in both age groups compared with the period before vaccine rollout (Figure 4: Appendix Table 5). Inequalities were still noticeable among those ≥65 years of age; HR was 1.08 (95% CI 1.02–1.14] for those living in Q2 areas, 1.09 (95% CI 1.03–1.15) in Q3 areas, 1.10 (95% CI 1.03–1.16) in Q4 areas, and 1.23 (95% CI 1.16–1.31) in Q5 areas. Conversely, among those 40–64 years of age, only those living in the most deprived areas had higher rates of infection (Q5 HR 1.04 [95% CI 1.00–1.08]). Regarding hospitalizations, inequalities by SDI quintile remained in both age groups, although they decreased among those ≥65 years of age: HR was 1.17 (95% CI 1.04–1.32) for those living in Q2 areas, 1.27 (95% CI 1.14–1.43) in Q3 areas, 1.29 (95% CI 1.15–1.45) in Q4 areas, and 1.52 (95% CI 1.36–1.71) in Q5 areas. Similarly, rates of COVID-19-related deaths among those >65 years of age in Q5 areas moderately decreased; HR was 1.36 (95% CI 1.02–1.82).

In sensitivity analyses restricting participants to citizens of Spain, results were also consistent with our main analyses (Appendix Figure 4). In the period 3–6 months after vaccine rollout, results were overall similar to our main analysis, although among those >65 years of age, inequalities in hospitalizations were more apparent than 1–6 months after vaccine rollout. HR for hospitalizations 3–6 months after vaccine rollout were 1.33 (95% CI 1.10–1.60) for those living in Q2 areas, 1.47 (95% CI 1.23–1.77 in Q3 areas, 1.42 (95% CI 1.18–1.71) in Q4 areas, and 1.71 (95% CI 1.42–2.06) in Q5 areas (Appendix Table 6).

## Discussion

In this cohort study comprising >2 million adults living in urban areas of Catalonia, Spain, vaccination coverage was high (>80%) 6 months after the COVID-19 vaccine rollout. However, coverage differed by SDI quintile for place of residence; coverage was 85% in the least deprived areas and 77% in the most deprived areas. Among retirement-age persons (>65 years), SDI quintile was not associated with vaccination, whereas among working-age persons (40–64 years), nonvaccination increased among those living in more deprived areas. Three months before vaccine rollout, we found a pattern of increased rates of COVID-19 infection and hospitalization among retirement-age and working-age persons living in more deprived areas. However, 6 months after rollout, socioeconomic inequalities in COVID-19 infection substantially decreased among both age groups, whereas inequalities in COVID-19 hospitalization moderately decreased only among retirement-age persons.

Surveys assessing inequalities in willingness to vaccinate (mostly conducted before vaccine rollout or shortly after) found conflicting results across countries (*21*–*23*). A study of 13,000 participants from 19 countries reported that younger age was associated with less willingness to vaccinate in the United Kingdom, Sweden, and Spain, whereas the opposite was observed in China (*22*). Conversely, higher education levels were associated with more willingness to vaccinate in the United States, France, and Germany, but not in Spain or the United Kingdom (*23*). Regarding COVID-19 vaccination coverage, studies are mostly limited to the United Kingdom (*7*,*24*,*25*) and the United States (*10*,*26*,*27*). However, these studies consistently found lower vaccination rates among persons with low socioeconomic status (*7*,*10*,*24*–*27*). This finding is also in line with prior evidence in relation to other vaccines (*28*,*29*). We found an association between higher socioeconomic deprivation and nonvaccination only among working-age persons. Differences by age group could be related to working conditions (i.e., unavailability to miss work to vaccinate), as well as to an enhanced COVID-19 risk perception among older persons, who have a higher risk for severe disease (*22*,*30*). Unlike our study, UK studies also observed inequalities in coverage among the elderly (*7*,*25*). Differences in the development of the pandemic, the vaccination campaign, or cultural perspectives across countries might explain these discrepancies. Spain was severely hit by the first wave of the pandemic (*17*) and is one of the countries with the highest COVID-19 vaccination coverages (*31*). Furthermore, Spain is a country with traditionally high levels of vaccine confidence and with high vaccination coverages overall (*32*).

Inequalities among working-age persons are concerning, because those with low socioeconomic status are more likely to be exposed to infection because of poorer working and housing conditions and to develop severe disease because of poorer health status (*4*,*33*). Those findings are consistent with our findings before vaccine rollout, as well as with prior evidence from the United States and Europe, including Spain (*3*,*34*,*35*). In July–November 2020 the risk ratio of COVID-19 infection in residents of the poorest areas of Barcelona, the capital of Catalonia, was 1.67 (95% CI 1.41–196) in men and 1.71 (95% CI 1.44–1.99) in women, in line with our findings (*35*).

Despite inequalities in vaccination coverage, socioeconomic inequalities for COVID-19 infection decreased 6 months after vaccine rollout among both age groups, suggesting that vaccines reduced inequalities partly through mechanisms of herd immunity (*36*). Conversely, inequalities in hospitalizations decreased, although they still persisted, only among retirement-age persons. This finding highlights the importance of addressing vaccine inequalities among working-age persons. Persisting inequalities among the retirement-age persons might be related to differences in the risk for severe COVID-19 once infected because we found that those living in more deprived areas have more comorbidities and, thus, higher risk for complications (*33*). In addition to nationwide vaccination campaigns, strategies addressing structural inequalities are needed to reduce the burden of COVID-19–related outcomes among those most vulnerable (*6*).

The main strength of this study is the nature of our database, which encompasses ≈75% of the population of Catalonia. In addition, our data include a complete record of vaccines administered and of COVID-19 tests performed at public healthcare facilities. This study provides novel evidence regarding the associations between socioeconomic deprivation and COVID-19 infection, hospitalization, and death before and after the COVID-19 vaccine rollout in a country in southern Europe.

The first limitation of our study is that, although area-based indices of socioeconomic deprivation are widely used in epidemiologic studies, our results should be interpreted with caution considering the risks of ecologic bias. Second, we lacked information on occupation, which would have been of interest to have a better understanding of our results among working-age persons; a UK study reported lower vaccination coverage among persons working in manual occupations (*37*). Last, our results might not be generalizable to other contexts because of differences across countries, although they provide insights into the effects on socioeconomic COVID-19 inequalities of a mass vaccination campaign in a high-income country with high access to vaccination.

Despite socioeconomic inequalities in vaccination coverage, our results show that inequalities in COVID-19 infection and hospitalization in urban areas decreased but still persisted 6 months after the start of vaccine rollout in Catalonia. Our findings show that mass COVID-19 vaccination reduced COVID-19-related inequalities and emphasize the need to pursue efforts to vaccinate all population subgroups.

Appendix Additional information about socioeconomic inequalities in COVID-19 vaccination and infection in adults, Catalonia, Spain, 2020–2021.
