# Supplementary material for: Socioeconomic Inequalities in COVID-19 Vaccination and Infection in Adults, Catalonia, Spain
Source: Emerg Infect Dis. 2022 Nov;28(11):2243–52. doi: 10.3201/eid2811.220614 (PMC9622244; doi:10.3201/eid2811.220614)
Supplement: Appendix — Additional information about socioeconomic inequalities in COVID-19 vaccination and infection in adults, Catalonia, Spain, 2020–2021. [file 22-0614-Techapp-s1.pdf]

Article DOI: https://doi.org/10.3201/eid2811.220614

Socioeconomic Inequalities in COVID-19 Vaccination and Infection in Adults, Catalonia, Spain

Appendix

Appendix Table 1. SNOMED codes used to identify baseline comorbidities and COVID-19 diagnoses prior to study start in study of socioeconomic inequalities in COVID-19 vaccinations and infection*

Condition	SNOMED code
Autoimmune disease	434621
Asthma	317009
Chronic obstructive pulmonary disease	255573
COVID-19 diagnoses	27619001
	186747009
	398447004
	441590008
	700217006
	713084008
	840539006
	840544004
Dementia	4182210
Heart disease	321588
Hypertensive disorder	316866
Malignant neoplastic disease	443392
Obesity*	433736
Renal impairment	4030518
Type 2 diabetes	201826

*Obesity was defined as having a record of a diagnosis of obesity (i.e., SNOMED code: 433736) and/or a body mass index (BMI) measurement ≥30 kg/m2.

Appendix Table 2. Baseline characteristics of the population included in a study of socioeconomic inequality in COVID-19 vaccination and outcomes as of September 1, 2020 by Socioeconomic Deprivation Index quintile

Characteristic	Quintile 1, least deprived (column %)	Quintile 2 (column %)	Quintile 3 (column %)	Quintile 4 (column %)	Quintile 5, most deprived (column %)
N	478,380	469,833	465,245	453,924	429,764
Loss to follow-up	8,359 (1.7)	6,725 (1.4)	7,247 (1.6)	7,513 (1.7)	8,436 (2.0)
Age in years, median (IQR)	58 (49, 70)	57 (48, 69)	57 (48, 70)	57 (47, 69)	55 (47, 68)
Age category					
40–49	130,288 (27.2)	139,359 (29.7)	140,531 (30.2)	141,235 (31.1)	143,511 (33.4)
50–59	120,820 (25.3)	119,532 (25.4)	116,265 (25.0)	113,462 (25.0)	112,479 (26.2)
60–69	102,390 (21.4)	94,064 (20.0)	91,005 (19.6)	86,017 (18.9)	76,697 (17.8)
70–79	75,548 (15.8)	70,643 (15.0)	71,613 (15.4)	69,149 (15.2)	58,199 (13.5)
≥80	49,334 (10.3)	46,235 (9.8)	45,831 (9.9)	44,061 (9.7)	38,878 (9.0)
Sex					
F	257,759 (53.9)	248,911 (53.0)	243,035 (52.2)	235,721 (51.9)	214,870 (50.0)
M	220,621 (46.1)	220,922 (47.0)	222,210 (47.8)	218,203 (48.1)	214,894 (50.0)
Nationality					
Spain	440,839 (92.2)	429,076 (91.3)	419,655 (90.2)	399,375 (88.0)	351,185 (81.7)
Africa	4,024 (0.8)	8,329 (1.8)	11,483 (2.5)	15,879 (3.5)	29,371 (6.8)
Central & South America	11,606 (2.4)	12,329 (2.6)	13,076 (2.8)	16,080 (3.5)	17,221 (4.0)
Asia & Oceania	5,756 (1.2)	6,392 (1.4)	6,863 (1.5)	9,324 (2.1)	19,571 (4.6)
Eastern Europe	4,616 (1.0)	6,772 (1.4)	7,729 (1.7)	7,891 (1.7)	7,795 (1.8)
Western Europe & North America	11,539 (2.4)	6,935 (1.5)	6,439 (1.4)	5,375 (1.2)	4,621 (1.1)
Comorbidities					
Asthma	26,307 (5.5)	28,492 (6.1)	28,800 (6.2)	29,488 (6.5)	28,638 (6.7)

Characteristic	Quintile 1, least deprived (column %)	Quintile 2 (column %)	Quintile 3 (column %)	Quintile 4 (column %)	Quintile 5, most deprived (column %)
Autoimmune disease	12,153 (2.5)	12,049 (2.6)	11,986 (2.6)	11,498 (2.5)	10,460 (2.4)
COPD	20,076 (4.2)	22,625 (4.8)	24,385 (5.2)	25,832 (5.7)	26,927 (6.3)
Dementia	6,078 (1.3)	5,937 (1.3)	6,281 (1.4)	6,153 (1.4)	5,774 (1.3)
Heart disease	79,325 (16.6)	81,047 (17.3)	83,056 (17.9)	82,116 (18.1)	76,845 (17.9)
Hypertension	148,744 (31.1)	155,270 (33.0)	160,986 (34.6)	159,831 (35.2)	150,589 (35.0)
Malignant neoplastic disease	59,704 (12.5)	55,590 (11.8)	53,564 (11.5)	51,138 (11.3)	44,662 (10.4)
Obesity	77,986 (16.3)	97,469 (20.7)	107,430 (23.1)	115,411 (25.4)	117,213 (27.3)
Renal impairment	30,960 (6.5)	32,886 (7.0)	35,652 (7.7)	35,879 (7.9)	34,570 (8.0)
Type 2 diabetes	46,020 (9.6)	54,110 (11.5)	59,669 (12.8)	63,074 (13.9)	65,315 (15.2)

*COPD, chronic obstructive pulmonary disease; IQR, interquartile range.

Appendix Table 3. Baseline characteristics of study participants with and without missing data on Socioeconomic Deprivation Index as of September 1, 2020

Characteristic	Without missing data (column %)	With missing data (column %)	SMD
N	2,297,146	307,038	
Age, median [IQR]	57 [48.0, 69.0]	55 [46.0, 68.0]	0.067
Age, categories			0.147
40–49	694,924 (30.3)	109,068 (35.5)	
50–59	582,558 (25.4)	75,154 (24.5)	
60–69	450,173 (19.6)	52,794 (17.2)	
70–79	345,152 (15.0)	36,076 (11.7)	
≥80	224,339 (9.8)	33,946 (11.1)	
Sex, male	1,096,850 (47.7)	153,851 (50.1)	0.047
Nationality			0.101
Spain	2,040,130 (88.8)	274,292 (89.3)	
Africa	69,086 (3.0)	7,917 (2.6)	
Central & South America	70,312 (3.1)	8,329 (2.7)	
Asia & Oceania	47,906 (2.1)	3,665 (1.2)	
Eastern Europe	34,803 (1.5)	5,512 (1.8)	
Europe & North America	34,909 (1.5)	7,323 (2.4)	
Comorbidities			
Asthma	141,725 (6.2)	18,846 (6.1)	0.001
Autoimmune disease	58,146 (2.5)	7,495 (2.4)	0.006
COPD	119,845 (5.2)	16,352 (5.3)	0.005
Dementia	30,223 (1.3)	7,899 (2.6)	0.091
Heart disease	402,389 (17.5)	53,903 (17.6)	0.001
Hypertension	775,420 (33.8)	95,514 (31.1)	0.057
Malignant neoplastic disease	264,658 (11.5)	35,211 (11.5)	0.002
Obesity	62,339 (20.3)	515,509 (22.4)	0.052
Renal impairment	169,947 (7.4)	25,002 (8.1)	0.028
Type 2 diabetes	35,561 (11.6)	288,188 (12.5)	0.030

*COPD, chronic obstructive pulmonary disease; IQR, interquartile range; SMD, standardized mean difference.

Appendix Table 4. Characteristics of persons with and without complete follow-up

Characteristic	With complete follow-up (column %)	Without complete follow-up (column %)	SMD
N	2,258,866	38,280	
Death for any cause	140 (0.0)	19,701 (51.5)	1.456
Characteristics as of 1 September 2020			
Age, median [IQR]	57 [48.0, 69.0]	69 [51.0, 84.0]	0.564
Age category			0.637
40–49	686,628 (30.4)	8,296 (21.7)	
50–59	576,770 (25.5)	5,788 (15.1)	
60–69	444,587 (19.7)	5,586 (14.6)	
70–79	339,351 (15.0)	5,801 (15.2)	
≥ 80	211,530 (9.4)	12,809 (33.5)	
Sex, male	1,076,273 (47.6)	20,577 (53.8)	0.122
Nationality			0.244
Spain	2,009,390 (89.0)	30,740 (80.3)	
Africa	67,135 (3.0)	1,951 (5.1)	
Central & South America	68,031 (3.0)	2,281 (6.0)	
Asia & Oceania	46,634 (2.1)	1,272 (3.3)	
Eastern Europe	33,869 (1.5)	934 (2.4)	
Europe & North America	33,807 (1.5)	1,102 (2.9)	
Socioeconomic Deprivation Index quintile			0.108
Quintile 1 (least deprived)	470,021 (20.8)	8,359 (21.8)	
Quintile 2	463,108 (20.5)	6,725 (17.6)	
Quintile 3	457,998 (20.3)	7,247 (18.9)	
Quintile 4	446,411 (19.8)	7,513 (19.6)	
Quintile 5 (most deprived)	421,328 (18.7)	8,436 (22.0)	
Comorbidities			
Asthma	139,615 (6.2)	2,110 (5.5)	0.029
Autoimmune disease	57,035 (2.5)	1,111 (2.9)	0.023
COPD	115,179 (5.1)	4,666 (12.2)	0.254
Dementia	26,693 (1.2)	3,530 (9.2)	0.368
Heart disease	389,357 (17.2)	13,032 (34.0)	0.392
Hypertension	757,215 (33.5)	18,205 (47.6)	0.289
Malignant neoplastic disease	255,354 (11.3)	9,304 (24.3)	0.345
Obesity	507,413 (22.5)	8,096 (21.1)	0.032
Renal impairment	161,567 (7.2)	8,380 (21.9)	0.428
Type 2 diabetes	279,905 (12.4)	8,283 (21.6)	0.248

*COPD, chronic obstructive pulmonary disease; IQR, interquartile range; SMD, standardized mean difference.

Appendix Table 5. Number of events, and crude and adjusted Hazard Ratios with 95%CI of COVID-19 infection, hospitalization and death before and after vaccine rollout by Socioeconomic Deprivation Index quintile, stratified by age group*

Age	Time period	Socioeconomic Deprivation Index quintile	N	Infections: Events (row %)	Infections: HR (95% CI) Crude	Infections: HR (95% CI) Adjusted	Hospitalizations: Events (row %)	Hospitalizations: HR (95% CI) Crude	Hospitalizations: HR (95% CI) Adjusted	Deaths: Events (row %)	Deaths: HR (95% CI) Crude	Deaths: HR (95% CI) Adjusted
≥65 y	3 mo before vaccine rollout	Q1, least deprived	173,037	3005 (1.7)	Reference	Reference	634 (0.4)	Reference	Reference	128 (0.1)	Reference	Reference
		Q2	160,220	3133 (2.0)	1.13 (1.07 to 1.18)	1.12 (1.07 to 1.18)	738 (0.5)	1.26 (1.13 to 1.40)	1.25 (1.12 to 1.39)	149 (0.1)	1.26 (0.99 to 1.59)	1.29 (1.02 to 1.64)
		Q3	160,219	3318 (2.1)	1.19 (1.14 to 1.25)	1.19 (1.13 to 1.25)	808 (0.5)	1.38 (1.24 to 1.53)	1.37 (1.23 to 1.52)	143 (0.1)	1.21 (0.95 to 1.53)	1.28 (1.00 to 1.62)
		Q4	153,050	3373 (2.2)	1.27 (1.21 to 1.34)	1.26 (1.20 to 1.32)	869 (0.6)	1.55 (1.40 to 1.72)	1.53 (1.38 to 1.70)	129 (0.1)	1.14 (0.89 to 1.45)	1.22 (0.95 to 1.56)
		Q5, most deprived	131,769	3574 (2.7)	1.57 (1.50 to 1.65)	1.54 (1.46 to 1.61)	988 (0.7)	2.05 (1.86 to 2.27)	1.99 (1.80 to 2.19)	157 (0.1)	1.61 (1.28 to 2.03)	1.71 (1.36 to 2.17)
	1 to 6 mo after vaccine rollout	Q1, least deprived	166,230	2341 (1.4)	Reference	Reference	531 (0.3)	Reference	Reference	93 (0.1)	Reference	Reference
		Q2	153,906	2345 (1.5)	1.08 (1.02 to 1.15)	1.08 (1.02 to 1.14)	579 (0.4)	1.18 (1.05 to 1.32)	1.17 (1.04 to 1.32)	81 (0.1)	0.94 (0.70 to 1.27)	0.95 (0.71 to 1.28)
		Q3	153,514	2357 (1.5)	1.09 (1.03 to 1.16)	1.09 (1.03 to 1.15)	630 (0.4)	1.28 (1.14 to 1.44)	1.27 (1.14 to 1.43)	103 (0.1)	1.20 (0.91 to 1.59)	1.23 (0.93 to 1.63)
		Q4	146,330	2263 (1.5)	1.10 (1.04 to 1.17)	1.10 (1.03 to 1.16)	611 (0.4)	1.30 (1.16 to 1.46)	1.29 (1.15 to 1.45)	89 (0.1)	1.09 (0.81 to 1.46)	1.12 (0.84 to 1.50)
		Q5, most deprived	125,009	2177 (1.7)	1.24 (1.17 to 1.32)	1.23 (1.16 to 1.31)	615 (0.5)	1.54 (1.37 to 1.73)	1.52 (1.36 to 1.71)	91 (0.1)	1.31 (0.98 to 1.74)	1.36 (1.02 to 1.82)
40–64 y	3 mo before vaccine rollout	Q1, least deprived	305,343	7675 (2.5)	Reference	Reference	483 (0.2)	Reference	Reference	<5	Reference	Reference
		Q2	309,613	8260 (2.7)	1.06 (1.03 to 1.10)	1.05 (1.02 to 1.08)	568 (0.2)	1.16 (1.03 to 1.31)	1.15 (1.02 to 1.30)	9 (0)	Not estimated	Not estimated
		Q3	305,026	8679 (2.8)	1.13 (1.10 to 1.17)	1.11 (1.08 to 1.15)	587 (0.2)	1.22 (1.08 to 1.37)	1.20 (1.06 to 1.35)	9 (0)	Not estimated	Not estimated
		Q4	300,874	9122 (3.0)	1.21 (1.17 to 1.25)	1.17 (1.13 to 1.20)	695 (0.2)	1.46 (1.30 to 1.64)	1.40 (1.24 to 1.57)	17 (0)	Not estimated	Not estimated
		Q5, most deprived	297,995	10,702 (3.6)	1.44 (1.40 to 1.48)	1.35 (1.31 to 1.39)	934 (0.3)	1.99 (1.78 to 2.22)	1.75 (1.57 to 1.96)	16 (0)	Not estimated	Not estimated
	1 to 6 mo after	Q1, least deprived	291,837	6794 (2.3)	Reference	Reference	478 (0.2)	Reference	Reference	<5		

Age	Time period	Socioeconomic Deprivation Index quintile	N	Infections			Hospitalizations			Deaths		
				Events (row %)	HR (95% CI)		Events (row %)	HR (95% CI)		Events (row %)	HR (95% CI)	
					Crude	Adjusted		Crude	Adjusted		Crude	Adjusted
	vaccine rollout	Q2	295,904	6972 (2.4)	1.01 (0.98 to 1.05)	1.00 (0.97 to 1.04)	563 (0.2)	1.16 (1.03 to 1.31)	1.17 (1.03 to 1.32)	8 (0)	Not estimated	Not estimated
		Q3	290,769	6919 (2.4)	1.02 (0.99 to 1.06)	1.01 (0.98 to 1.05)	621 (0.2)	1.30 (1.16 to 1.47)	1.31 (1.16 to 1.47)	7 (0)	Not estimated	Not estimated
		Q4	285,830	6558 (2.3)	0.99 (0.95 to 1.02)	0.97 (0.94 to 1.00)	641 (0.2)	1.37 (1.22 to 1.54)	1.35 (1.20 to 1.53)	13 (0)	Not estimated	Not estimated
		Q5, most deprived	280,872	6900 (2.5)	1.06 (1.02 to 1.09)	1.04 (1.00 to 1.08)	697 (0.2)	1.52 (1.35 to 1.71)	1.46 (1.30 to 1.64)	<5	Not estimated	Not estimated

*Vaccine rollout started on 27 December 2020. Models before vaccine rollout are from 1 September to 26 December 2020, the median follow-up time was 117 d [IQR: 117–117] for all outcomes. Models after vaccine rollout are from 27 January to 30 June 2021, the median follow-up time was 154 d [154–154] for all outcomes. Adjusted models are adjusted for age, sex and nationality. Models in which the number of events was below 5 for some subgroup populations were not estimated. HR: Hazard Ratio; CI: Confidence Interval; Q:quintile.

Appendix Table 6. Number of events, and crude and adjusted Hazard Ratios with 95%CI of COVID-19 infection, hospitalization and death 3 to 6 mo after vaccine rollout by Socioeconomic Deprivation Index quintile, stratified by age group*

Age group	Socioeconomic Deprivation Index quintile	N	Infections			Hospitalizations			Deaths		
			Events (row %)	HR (95% CI)		Events (row %)	HR (95% CI)		Events (row %)	HR (95% CI)	
				Crude	Adjusted		Crude	Adjusted		Crude	Adjusted
≥65 y	Q1, least deprived	164,110	1009 (0.6)	Reference	Reference	199 (0.1)	Reference	Reference	32 (0)	Reference	Reference
	Q2	151,888	1027 (0.7)	1.10 (1.01 to 1.20)	1.10 (1.01 to 1.20)	243 (0.2)	1.33 (1.10 to 1.61)	1.33 (1.10 to 1.60)	27 (0)	0.91 (0.55 to 1.52)	0.91 (0.55 to 1.53)
	Q3	151,372	1041 (0.7)	1.12 (1.03 to 1.22)	1.11 (1.02 to 1.22)	270 (0.2)	1.49 (1.24 to 1.78)	1.47 (1.23 to 1.77)	30 (0)	1.02 (0.62 to 1.67)	1.03 (0.63 to 1.69)
	Q4	144,169	1006 (0.7)	1.13 (1.04 to 1.24)	1.13 (1.04 to 1.23)	248 (0.2)	1.43 (1.19 to 1.73)	1.42 (1.18 to 1.71)	35 (0)	1.24 (0.77 to 2.01)	1.26 (0.78 to 2.04)
	Q5, most deprived	122,690	916 (0.7)	1.22 (1.11 to 1.33)	1.20 (1.10 to 1.31)	258 (0.2)	1.74 (1.44 to 2.09)	1.71 (1.42 to 2.06)	28 (0)	1.17 (0.70 to 1.94)	1.20 (0.72 to 1.99)
40–64 y	Q1, least deprived	287,892	3336 (1.2)	Reference	Reference	225 (0.1)	Reference	Reference	<5		
	Q2	292,062	3514 (1.2)	1.04 (0.99 to 1.09)	1.03 (0.98 to 1.08)	283 (0.1)	1.23 (1.04 to 1.47)	1.23 (1.04 to 1.47)	5 (0)	Not estimated	Not estimated
	Q3	286,848	3420 (1.2)	1.03 (0.98 to 1.08)	1.01 (0.96 to 1.06)	307 (0.1)	1.37 (1.15 to 1.63)	1.36 (1.15 to 1.62)	<5	Not estimated	Not estimated
	Q4	281,988	3258 (1.2)	1.00 (0.95 to 1.05)	0.97 (0.93 to 1.02)	326 (0.1)	1.47 (1.24 to 1.75)	1.45 (1.22 to 1.72)	5 (0)	Not estimated	Not estimated
	Q5, most deprived	276,464	3331 (1.2)	1.04 (0.99 to 1.09)	1.00 (0.96 to 1.05)	344 (0.1)	1.59 (1.34 to 1.88)	1.51 (1.27 to 1.79)	<5	Not estimated	Not estimated

*Vaccine rollout started on 27 December 2020. Models are from 27 March to 30 June 2021, the median follow-up time was 95 d [IQR: 95–95] for all outcomes. Adjusted models are adjusted for age, sex and nationality. Models in which the number of events was below 5 for some subgroup populations were not estimated. HR, Hazard Ratio; Q, quintile.

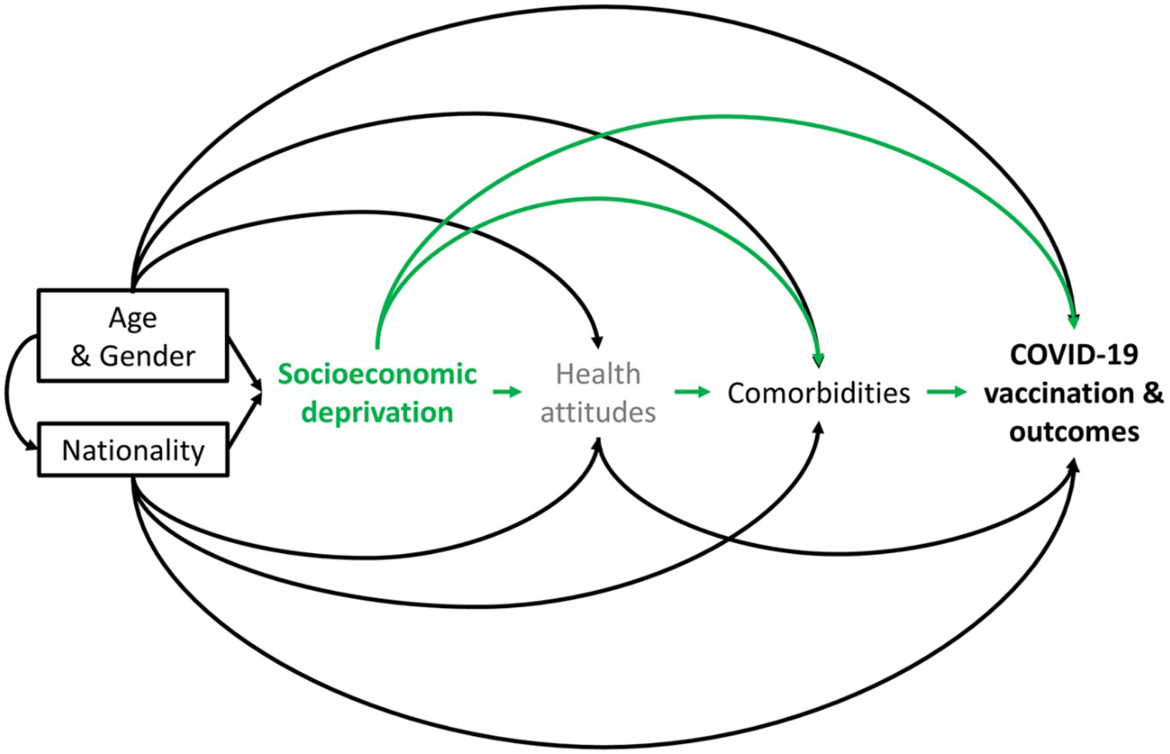

Appendix Figure 1. Directed acyclic graph used to adjust models assessing the relationship between socioeconomic deprivation and COVID-19 vaccination, infection, hospitalization, and death. We used sex as a proxy of gender. Socioeconomic deprivation was measured using a Socioeconomic Deprivation Index. COVID-19 outcomes include COVID-19 infections, hospitalizations, and deaths.

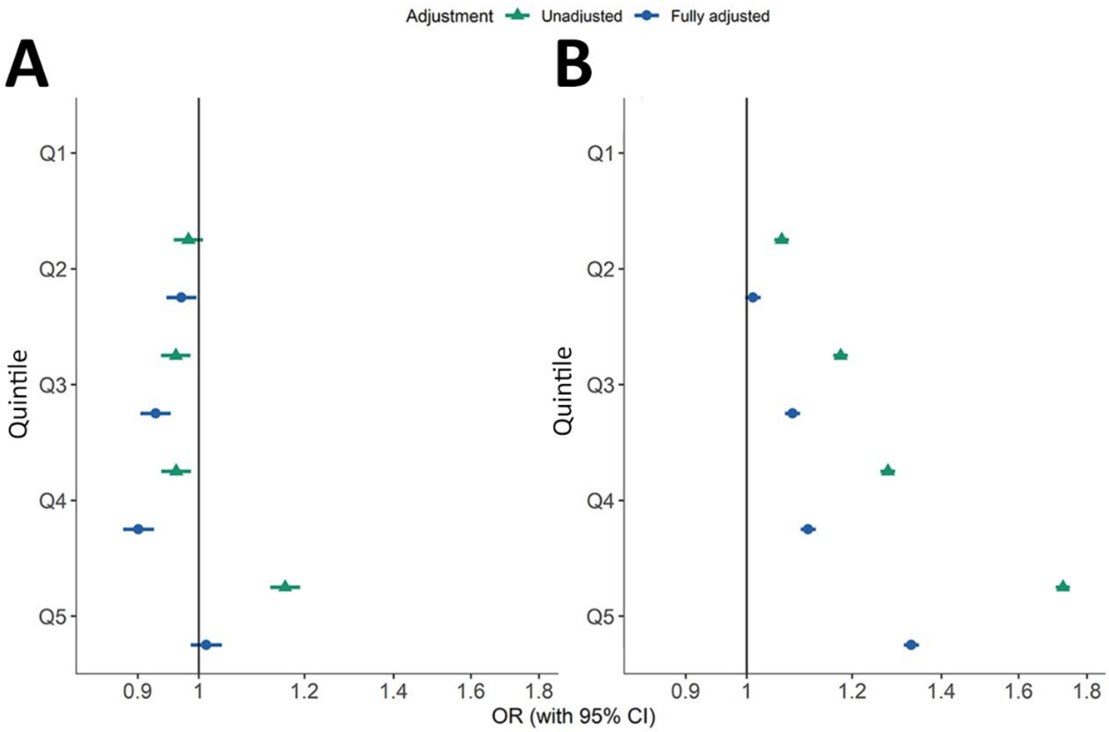

Appendix Figure 2. Unadjusted and fully adjusted odds ratios of nonvaccination for COVID-19 by Socioeconomic Deprivation Index quintile, stratified by age group. Fully adjusted models are adjusted for age, sex and nationality. OR, odds ratio; Q, quintile; Ref, referent. A) OR for retirement-age persons $\geq$65 years of age. B) OR for working-age persons 40–64 years of age.

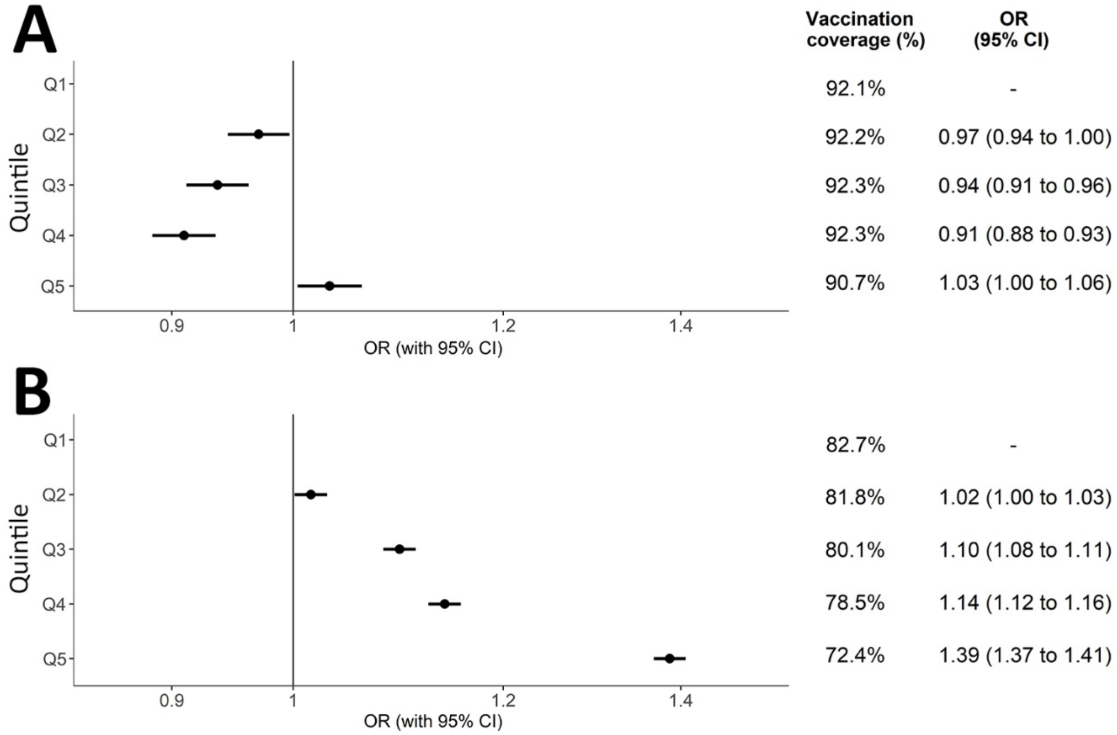

Appendix Figure 3. Odds ratios of nonvaccination for COVID-19 by Socioeconomic Deprivation Index quintile, stratified by age group after excluding persons with a COVID-19 infection before vaccination during study follow-up. Models adjusted for age, sex and nationality. OR, odds ratio; Q, quintile; Ref, referent. A) OR for retirement-age persons $\geq$65 years of age. B) OR for working-age persons 40–64 years of age.

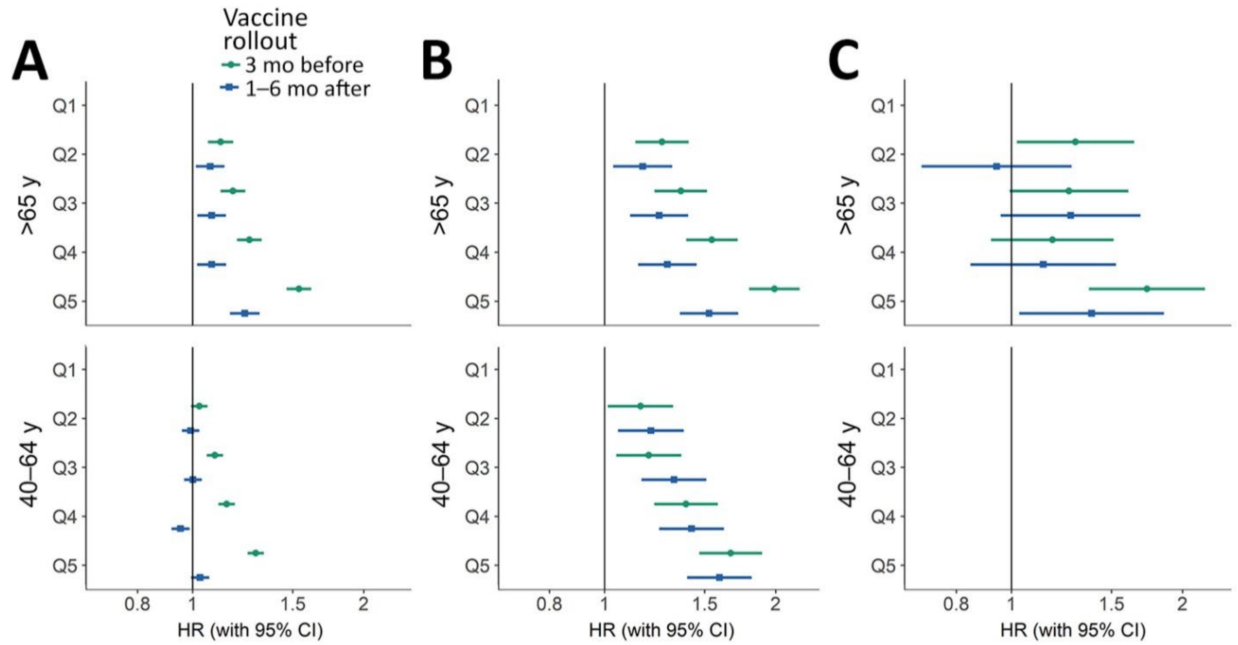

Appendix Figure 4. Fully adjusted hazard ratios of COVID-19 infection (A), hospitalization (B), and death (C) among Spanish citizens before and after vaccine rollout, by Socioeconomic Deprivation Index quintile and stratified by age group. Vaccine rollout started on December 27, 2020. Models before vaccine rollout (n = 2,040,130) are from September 1–December 26, 2020, the median follow-up time was 117 days (IQR 117–117) for all outcomes. Models after vaccine rollout (n = 1,950,130) are from 27 January to 30 June 2021, the median follow-up time was 154 days (IQR 154–154) for all outcomes. Adjusted models are adjusted for age, sex and nationality. Models in which the number of events was below 5 for some subgroup populations were not estimated. HR, hazard ratio; Q, quintile, Ref, reference.
